# Dietary Patterns during Pregnancy and Gestational Weight Gain: A Systematic Review

**DOI:** 10.1055/s-0042-1744290

**Published:** 2022-04-28

**Authors:** Larissa Bueno Ferreira, Cecília Viana Lobo, Aline Elizabeth da Silva Miranda, Brenda da Cunha Carvalho, Luana Caroline dos Santos

**Affiliations:** 1 Child and Adolescent Health, Federal University of Minas Gerais, Faculdade de Medicina/UFMG, Belo Horizonte, MG, Brazil; 2 Nutrition Departament, Federal University of Minas Gerais, Escola de Enfermagem/UFMG, Belo Horizonte, MG, Brazil

**Keywords:** gestational dietary patterns, weight gain, pregnancy, pregnant women, diets, padrões alimentares gestacionais, ganho de peso, gravidez, gestantes, dietas

## Abstract

The present systematic review (PROSPERO: CRD42020148630) hypothesizes the association of excessive weight gain during pregnancy with dietary patterns composed of ultraprocessed foods. Thus, the objective was to investigate the association between dietary patterns after analysis and weight gain during pregnancy. The search for articles was performed in nine databases. Two reviewers selected the articles in the databases and extracted from them the data used in the review. Two scales were used to evaluate the quality of the selected studies: New Castle-Ottawa Quality Assessment for cohort-based studies and Appraisal tool for Cross-Sectional Studies (AXIS) for cross-sectional-based studies. In total, 11 studies were identified with sample size variation ( *n* = 173–5,733). Women presenting more adherence to healthy and traditional patterns (fruits, vegetables, salads, nuts, and dairy) recorded less excessive gestational weight gain (GWG). Higher intake of mixed patterns and western patterns rich in ultraprocessed foods were associated with a higher prevalence of excessive GWG (24.48–55.20%). Gestational dietary patterns a posteriori *-* derived that have presented ultraprocessed components rich in fat and sugars presented association with high GWG; healthy and traditional dietary patterns were related to better mother-child health conditions, such as adequate GWG.

## Resumo

A presente revisão sistemática (PROSPERO: CRD42020148630) tem como hipótese que o ganho de peso excessivo durante a gravidez está associado aos padrões alimentares compostos por alimentos ultraprocessados. Desta forma, objetivou-se investigar a associação entre o padrão alimentar a posteriori e o ganho de peso durante a gestação. A busca de artigos foi realizada em nove bases de dados. Dois revisores selecionaram os artigos nestas bases e extraíram as informações utilizadas na revisão. Duas escalas foram utilizadas para avaliar a qualidade dos estudos selecionados: Escala de Avaliação da Qualidade de New Castle-Ottawa para estudos baseados em coortes e a Ferramenta de Avaliação de Estudos Transversais (escala AXIS) para estudos transversais. No total, foram identificados 11 trabalhos com variação do tamanho amostral ( *n* = 173–5.733). As mulheres que apresentaram maior adesão aos padrões alimentares saudáveis e tradicionais (frutas, hortaliças e vegetais, nozes e laticínios) apresentaram menor ganho de peso gestacional (GPG). A maior ingestão de padrões alimentares mistos e ocidentais ricos em alimentos ultraprocessados foi associada a uma maior prevalência de GPG excessivo (24,48–55,20%). Os padrões alimentares gestacionais derivados a posteriori que apresentaram componentes ultraprocessados ricos em gordura e açúcares apresentaram associação com o maior GPG; os padrões alimentares saudáveis e tradicionais foram relacionados a melhores condições de saúde materno-infantil, como GPG adequado.

## Introduction

Dietary patterns capture the interaction and cumulative effect of various foods and nutrients and can be easily interpreted by the population and, therefore, are particularly important in public health. It is important to note that people do not eat isolated nutrients. Instead, they eat meals that consist of a variety of foods with complex combinations of nutrients that are likely to be interactive or synergistic. 1

Dietary patterns can be based on indices, assessed a priori using dietary indices to measure adherence to a predefined dietary pattern, or data-driven—assessed a posteriori, in which dietary patterns are statistically derived based on food intake reported by a population. 2

The *a posteriori* method is considered more robust, making it possible to find the real dietary patterns of the study population, without making any assumption of protection or harmful effects on health. 3

Maternal nutrition during pregnancy is an important determinant for both maternal and infant outcomes. The examination of dietary patterns emerged as a more holistic approach to capture the complex interactions between nutrients and food, congruent with the dietary guidelines adopted by health agencies and international references. 4

Among the outcomes of the pregnancy period, gestational weight gain (GWG) is an important predictor of adverse maternal and child health outcomes. Inadequate or excessive weight gain can lead to undesirable health conditions for the mother or children, 5 with different prevalences of inadequate weight gain among populations. In the United States, only 32% of women who give birth to babies at term meet GWG recommendations of the Institute of Medicine (IOM). 6

Inadequate GWG is related to preterm birth, low weight at birth, and difficulty to start breastfeeding. 4 5 7 Moreover, excessive weight gain is associated with unfavorable outcomes such as gestational diabetes, gestational high blood pressure, cesarean section surgery, and child obesity. 7 8 9 In addition, a systematic review and meta-analysis 10 found that excessive GWG was also associated with both cesarean surgery and fetal macrosomia. Besides, excessive GWG helps worsening the global obesity outbreak, a fact that can lead to a great economic burden in both developed and developing countries. 5

Different factors can influence weight gain, with an emphasis on the eating patterns of the mother throughout pregnancy. Studies show an association between dietary patterns and GWG in western populations. 11 12

Therefore, the most likely hypothesis is that excessive weight gain during pregnancy is associated with dietary patterns composed of ultraprocessed foods.

Thus, the aim of the present study is to investigate the association between a posteriori dietary patterns and analysis of weight gain during pregnancy. In addition, the present study aims to update the practices of health professionals to improve care during pregnancy. This makes room for a better understanding of the association between dietary patterns and better health outcomes.

## Methods

A preliminary search was performed in the following databases: Medical Literature Analysis and Retrieval System Online (MEDLINE) through PubMed, PROSPERO, and Cochrane Library to assure article authenticity, and no reviews about the topic were found. The Preferred Reporting Items for Systematic Reviews and Meta-Analysis Protocol (PRISMA) were adopted. 13

Nine databases were searched, including the MEDLINE, Cumulative Index to Nursing and Allied Health Literature (CINAHL), Scopus, Web of Science, Virtual Health Library (BVS, in the Portuguese acronym), Latin American and Caribbean Health Sciences Literature (LILACS), Spanish Bibliographic Index of Health Sciences (IBECS), Scientific Eletronic Library Online (SciELO), and SciFinder databases.

The following terms, words, and combinations of words were searched: ( *dietary patterns* OR *dietary intake patterns* OR *patterns of food consumption* OR *food profile* AND *pregnancy* OR *pregnant women* OR *gravid* OR *gestation* AND *gestational weight gain* OR *weight gain* OR *postpartum period* ), as well as its translations into Spanish and Portuguese. The PROSPERO registration was performed under number CRD42020148630.

The studies were screened by title and then by abstract by two reviewers. The full texts of all selected studies were critically reviewed based on the inclusion and exclusion criteria. The inclusion criteria considered for the present review study were: (a) original articles; (b) studies using a posteriori dietary patterns derived as exposure variable and GWG as outcome variable; and (c) published between 2009 and January 2021. The exclusion criteria were: (a) articles that examined only individual nutrients or foods; (b) articles that used a priori dietary pattern analysis; (c) articles that featured dietary patterns concerning periods other than pregnancy; (d) duplicate articles in the databases; (e) experimental and animal studies.

The quality of the selected full-text articles was rated by two reviewers independently using the New Castle-Ottawa Quality Assessment for cohort-based studies and the Appraisal tool for Cross-Sectional Studies (AXIS) for cross-sectional-related studies.

The New Castle-Ottawa Quality Assessment scale assesses eight study items divided into three domains: selection, comparability, and outcome. The scoring system ranges from 0 to n stars – ≥ 6 stars are considered good scores. The AXIS scale takes into account 20 items, which are divided into 5 domains: introduction, methods, results, discussion, and others. Scores in the AXIS scale range from 0 to 20 points, and scores – ≥ 15 points are considered good. Other authors adopted similar cutoff points. 14 15 16

All articles used in the present review recorded good scores ( Chart 1 ); therefore, they were considered of good quality.

**Chart 1:** Features of articles included in the systematic review

Authors, year	City/Country	Design	Sample (n)	Instruments used for quality evaluation	Score*
Wei et al. (2019) 5	China	Cohort	5733	New Castle-Ottawa Quality Assessment Scale	*********
Suliga et al. (2018) 6	Poland	Cross-sectional	458	Appraisal tool for Cross-Sectional Studies (AXIS)	16
Alves-Santos et al. (2018) 11	Rio de Janeiro, Brazil	Cohort	173	New Castle-Ottawa Quality Assessment Scale	********
Shin et al. (2016) 12	United States	Cross-sectional	391	Appraisal tool for Cross-Sectional Studies (AXIS)	19
Uusitalo et al. (2009) 17	Finland	Cohort	3360	New Castle-Ottawa Quality Assessment Scale	*********
Tielemans et al. (2015) 18	Netherlands	Cohort	3374	New Castle-Ottawa Quality Assessment Scale	*********
Maugeri et al. (2019) 20	Catania, Italy	Cohort	232	New Castle-Ottawa Quality Assessment Scale	******
Cano-Ibáñez et al. (2020) 21	Spain	Cohort	533	New Castle-Ottawa Quality Assessment Scale	*********
Wrottesley et al. (2017) 22	South Africa	Cohort	538	New Castle-Ottawa Quality Assessment Scale	*******
Angali et al. (2020) 23	Iran	Cohort	488	New Castle-Ottawa Quality Assessment Scale	********
Itani et al. (2020) 24	United Arab Emirates	Cohort	242	New Castle-Ottawa Quality Assessment Scale	********

Note: *New Castle-Ottawa Quality Assessment Scale: score, 0 to 9 stars – 6 stars, or more, were considered good scores; Appraisal tool for Cross-Sectional Studies (AXIS): score, 0 to 20–15 points, or more, were considered good scores

The data were entered into a Microsoft Excel, version 16 (Microsoft Corporation, Redmond, WA, USA) spreadsheet and exported to the IBM SPSS Statistics for Windows, version 19.0 (IBM Corp., Armonk, NY, USA). Article inclusion and data extraction were made in an independent way; result comparisons were performed through the Kappa test. Disagreements were solved by consensus between reviewers—a third reviewer should be requested in case of disagreement between peers.

The following information were extracted: authors, publication year, city and country, study design, sample size, method to identify dietary patterns, dietary patterns identified, main results, and inadequate and/or excessive GWG prevalence.

## Results

**Fig. 1:**
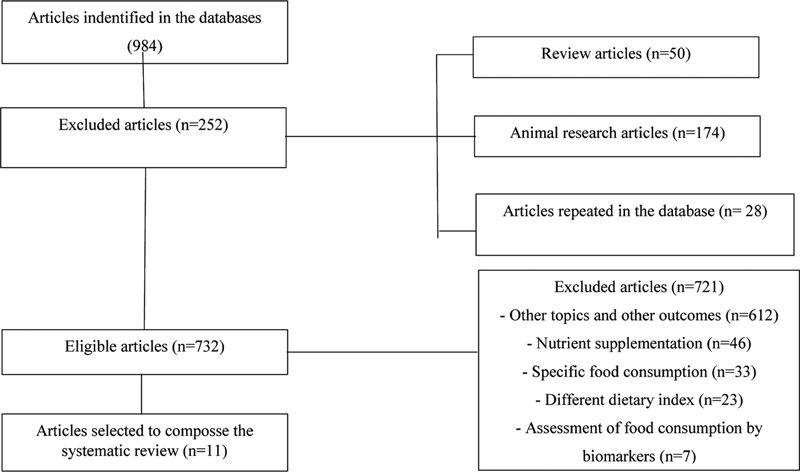
Flowchart describing the article-selection process for the systematic review.

We identified 984 articles, 973 (98.88%) of which were considered unsuitable for the preparation of the present material. For the present review, 11 articles addressing dietary patterns and GWG were considered eligible ( Fig. 1 ). The Kappa test result (0.887) pointed toward excellent agreement between reviewers.

Five studies were performed in Europe, 6 17 18 19 20 21 one in North America, 19 one in South America, 11 one in South Africa, 22 and three in Asia. 5 23 24 Nine (81.82%) of the 11 assessed studies followed a cohort-based design to find the assessed data, whereas 2 of them (18.18%) were cross-sectional-based studies. The samples in these studies ranged from 173 to 5,733 participants ( Chart 1 ).

All studies selected to compose the present review have used a food frequency questionnaire (FFQ) to assess the food intake of women. A posteriori dietary patterns were derived out through principal components analysis (PCA) on the majority ( *n* = 9). One study used Clusters analysis to determinate the patterns, and another study used reduced rank regression (RRR) ( Chart 2 ).

**Chart 2:** Identified dietary patterns, main results, and inadequate and/or excessive gestational weight gain prevalence

Authors, years	Dietary patterns method	Identified dietary patterns	Main results	Excessive GWG	Inadequate GWG
Suliga et al. (2018) 6	PCA	“Prudent” “Varied” “Unhealthy”	Prudent Women with excessive GWG presented less adherence to this pattern (OR 0.47; *p* = 0.033)	32.97%	21.83%
Alves-Santos et al. (2018) 11	RRR	“Common- Brazilian” “Western”	There was no association between the identified dietary patterns and GWG	34.68%	*
Shin et al. (2016) 12	PCA	“Healthy” “Mixed” “Western”	Mixed Higher adherence to this pattern was associated with greater odds of inadequate GWG when compared with lower adherence (AOR 4.72 95%CI 1.07–20.94)	51.15%	28.39%
Uusitalo et al. (2009) 17	PCA	“Healthy” “Fast Food” “Traditional bread” “Traditional meat” “Low-fat” “Coffee” “Alcohol and Butter”	Fast food pattern Positively associated with GWG rate (β = 0.010, *p* = 0.004) Alcohol and butter pattern Inversely associated with GWG rate (β = -0.010, *p* < 0.0001)	*	*
Tielemans et al. (2015) 18	PCA	“Vegetable, oil and fish” “Nuts, high-fiber cereals and soy” “Margarine, sugar and snacks”	Margarine, sugar and snacks Higher scores on this pattern resulted in a higher prevalence of excessive GWG (OR 1.45 95%CI 1.06–1.99)	24.48%	13.60%
Maugeri et al. (2019) 20	PCA	“Western” “Prudent”	Western The adherence to this pattern was associated with greater GWG (β = 1.217, *p* = 0.013)	27.59%	31.46%
Wrottesley et al. (2017) 22	PCA	“Traditional” “Mixed” “Western”	Mixed Significant and positive association with GWG rate (β = 22 *p* = 0.004)	55.20%	23.79%

Abbreviations: AOR, adjusted odds ratio; CI, confidence interval; GWG, gestational weight gain; OR, odds ratio; p: significance level; PCA, principal component analysis; RRR: reduced rank regression.

*Not informed by the study.

### Association between Food Patterns and Gestational Weight Gain

Seven studies showed positive associations between dietary patterns and GWG. A study performed by Uusitalo et al. 17 showed that two out of seven dietary patterns (fast-food and traditional breads) had positive association with the GWG rate. Only the fast-food pattern, rich in ultraprocessed foods like sweets, soft drinks, hamburgers, pizza, and other fast-foods, remained GWG-significant after the models were adjusted to all confounding factors, including maternal age at delivery, pregestational body mass index (BMI), parity, residence location, vocational education, smoking, and birthweight (β = 0.010; *p* = 0.004). 17

Tielemans et al. 18 presented a prevalence of 43% of women with excessive GWG. They did not find associations between higher adherence to the dietary patterns and GWG prevalence; however, women recording higher scores for the “margarine, sugar, and snacks” pattern had a higher prevalence of excessive GWG than the ones in the lowest quartile (odds ratio [OR] Q4: 1.45; 95% confidence interval [CI]: 1.06–1.99). This pattern was also significantly associated with higher weight in normal weight women (mean 0.30; 95%CI: 0.07–0.52; *p* < 0.05) throughout pregnancy.

Although Maugeri et al. 20 did not find associations between dietary patterns and GWG in the univariate analyses, they performed a linear regression model adjusted to age, weight at delivery, gestational duration, educational level, working status, smoking, parity, newborn gender, and total energy intake. This model showed a positive trend of GWG across tertiles of the western dietary pattern—high consumption of red meat, fries, dipping sauces, salty snacks, and alcoholic drinks (β = 1.217; se = 0.487; *p* = 0.013). They found no associations between excessive GWG and adherence to the prudent dietary pattern in the assessed population.

Wei et al. 5 found a prevalence of 31.3% of women with excessive GWG. The “richer in fish, beans, nuts, and yogurt” pattern was the one that registered the greater proportion of participants (23.2%), while the richer in fruits pattern registered the lowest proportion (11.2%). The “richer in fruits” pattern was positively correlated to GWG in both the total GWG and GWG rates. The other patterns did not present significant correlation with GWG. The “richer in fruits” pattern was associated with excessive GWG after adjustments to confounders such as maternal age, educational level, prepregnancy BMI, and parity.

Wrottesley et al. 22 found a prevalence of 55% of women presenting excessive GWG. In the total sample of pregnant women, only the “mixed” pattern (characterized by high consumption of grains, nuts, and dairy as well as added sugar and sweet spreads) showed significant and positive association with the GWG rate in both crude and adjusted models (adjusted to other patterns, parity, marital status, and total energy intake). This positive association was maintained in obese women for all models (Model 1: 25–11.4 g/week; *p* = 0.029; Model 2: 23–11.4 g/week; *p* = 0.042; Model 3: 24–11.6 g/week; *p* = 0.041) but was not observed in normal weight or overweight women.

The western pattern was significantly associated with a higher weight gain rate in normal weight women in all models. To the GWG category analysis, in crude logistic regression, a higher western diet pattern score was associated with increased odds of excessive weight gain in normal weight women.

In a study conducted with Emirati and Arab women, Angali et al. 23 identified two dietary patterns: “fast food with high fat” pattern, which included pasta, vermicelli, broken wheat, high-fat organ meats, high-fat dairy, sugary cool drinks, and ultraprocessed meats (salami and sausage), and the “vegetable, fruit, and protein” pattern. The higher the adherence to the “fast food with high fat” pattern, the covariance adjusted analysis and unadjusted multiple regression analysis indicated that the this pattern was a significant positive predictor of increase in GWG in the 1 ^st^ (adjusted b = 0.009; 95%CI: 0.001–0.017) and 3 ^rd^ trimester of pregnancy (adjusted b = 0.029; 95%CI: 0.012–0.049). On the other hand, in the fully adjusted quartile regression model, women in the highest quartile (Q99) of the “vegetable, fruit, and protein” pattern showed a negative and significant association (adjusted β = - 1; 95%CI: - 1.97–- 0.03).

Likewise, Itani et al. 24 found two dietary patterns, the *diverse* pattern, characterized by high inputs of fruits, vegetables, mixed dishes, meats, dairy products, grains, vegetables, and nuts, and the *western* pattern, rich in ultraprocessed foods (sweets, sugar-sweetened drinks, fast-food, and added sugars). The western pattern was associated with excessive GWG (OR: 4.04; 95%CI: 1.07–15.24) and GWG rate (OR: 4.38; 95%CI: 1.28–15.03), while the diverse pattern decreased the risk of inadequate GWG (OR: 0.24; 95%CI: 0.06–0.97) and GWG rate (OR: 0.28; 95%CI: 0.09–0.90).

Three studies found dietary patterns with protective effect in terms of GWG. Wrottesley et al. 22 showed that the *traditional* pattern (high in whole grains, legumes, vegetables, and traditional meat) had significant, inverse associations with the GWG rate in the crude model, and to parity and marital status adjusted (model 1: - 2 7; 11.1 g/week; *p* = 0.015; model 2: - 27; 11.5 g/week; *p* = 0.02); however, this association was no longer significant after adjustment for total energy intake. In line with these findings, the study conducted by Suliga et al. 6 showed that in women with excessive GWG, a lower adherence to the *prudent* pattern was noted in comparison with other participants in the study. The *prudent* pattern found in this study (high intake of whole grains, vegetables, legumes, sea fish, milk, and dairy products, and avoiding snacking between meals) is similar in terms of composition to the *traditional* pattern described by Wrottesley et al. 22

In addition, Cano-Ibáñez et al. 21 found moderate evidence for an association between the Mediterranean eating pattern, considered a healthy eating pattern (vegetables, olive oil, whole grains, and nuts) and lower GWG trajectories (0.06; 95%CI: - 0.11–- 0.04) and better nutrient adequacy.

One study found that the *mixed* pattern also showed significant associations with inadequate GWG. 12 In the physical activity level-adjusted model, women in the highest tertile of the *mixed* pattern (high intake of meat, dairy products, fruits, vegetables, potatoes, nuts and seeds, and sweets) had significantly greater odds of inadequate GWG when compared with those in the lowest tertile (AOR: 4.72; 95%CI:1.07–20.94). Women in the midtertile of the *mixed* pattern presented a lower OR of excessive GWG compared with those in the lowest tertiles (OR: 0.39; 95%CI: 0.15–0.99). The other patterns did not show significant GWG associations.

### Dietary Pattern Association and other Outcomes

The assessed studies also associated GWG and dietary patterns with other maternal and child health outcomes.

Suliga et al. 6 found in the crude model a positive association between an increased risk of excessive GWG and prepregnancy BMI ≥ 25 kg/m2 (OR = 6.44; *p* < 0.001) and with giving up smoking (OR = 9.07; *p* = 0.004). A lower risk of excessive GWG was associated with being underweight prepregnancy compared with having a normal BMI (OR = 0.17; *p* = 0.020). In the adjusted model, the factor increasing the risk of inadequate GWG was being underweight prepregnancy (OR= 2.61; *p* = 0.018), but this risk was significantly lower in the third, or subsequent, pregnancy compared with the first one (OR = 0.39; *p* = 0.042).

Maugeri et al. 20 showed that prepregnancy weight and BMI decreased across tertiles of the *prudent* dietary pattern ( *p* = 0.043 and *p* = 0.019, respectively). In fact, women presenting higher adherence to this pattern were less likely to be overweight or obese ( *p* = 0.007). Linear regression results confirmed the negative association between prepregnancy BMI and adherence to the *prudent* dietary pattern after adjustments regarding age, educational level, employment status, smoking, total energy intake, and gestational age at recruiting (β = - 0.631; se = 0.318; *p* = 0.038). Women in the 3 ^rd^ tertile of the *prudent* dietary pattern showed lower prepregnancy BMI than the ones in the 1 ^st^ tertile (β = - 1.347; se = 0.598; *p* = 0.024).

Angali et al. 23 identified that women with pregestational BMI > 25 kg/m ^2^ had more adherence to the “vegetable, fruit, and protein” pattern than those with low adherence (3 ^rd^ tercil versus 1 ^st^ tercil). On the other hand, women with normal weight and underweight showed a greater tendency to the *fast food with high fat* pattern (3 ^rd^ tercil versus 1 ^st^ tercil).

Wrottesley et al. 22 did not find any association between BMI in the 1 ^st^ gestational semester and the dietary patterns identified in their research.

The multiple adjusted longitudinal analyses conducted by Alves-Santos et al. 11 showed that higher adherence to the common-Brazilian dietary pattern was directly associated with adiponectin concentrations (β = 1.07; 95%CI: 0.17–1.98). On the other hand, highest adherence to the western dietary pattern was negatively associated with adiponectin throughout pregnancy (high versus low tertile of adherence β = - 1.11; 95%CI - 2.00–- 0.22) and directly associated with leptin concentrations (β = 64.9; 95%CI: 22.8–107.0).

Finally, Cano-Ibáñez et al. 21 identified that, regardless of the GWG, the Mediterranean dietary pattern showed moderate evidence of a greater likelihood of achieving an adequate dietary fiber intake, vitamins B9, D and E, and iodine ( *p* < 0.05).

## Discussion

To the best of our knowledge, this is the first review addressing dietary patterns a posteriori-derived and their association with GWG. The studies were performed especially in European and Asian countries, and the most used method was the principal components analysis. The high prevalence of inadequacy and/or excess of GWG (35.10 to 55.20%) is the last one confirming the hypothesis about the dietary pattern composed of ultraprocessed foods and its outcomes in the weight gain of pregnant woman, proves the importance of better understand the process, both in the health of women and children.

Dietary patterns are not exactly the same in studies; however, it is clear from published studies that certain dietary patterns like *western* / *unhealthy* , *healthy* / *prudent* / *Mediterranean,* and *traditional* are often found. 24 The assessed studies were similar in the association of dietary patterns that shows higher caloric density 12 18 19 22 24 with greater chances of excessive GWG outcomes, as well as patterns presenting healthier and more traditional components 6 21 22 being associated with lower GWG.

The *healthy* / *prudent* / *Mediterranean* dietary patterns were characterized by high consumption of whole grains, vegetables, legumes, sea fish, olive oil, and nuts. 6 21 Accordingly, the Dietary Guidelines for the Brazilian Population has, as one of its recommendations, that the base of a healthy diet should be in natura or minimally processed food, mostly from vegetal origin. 25 26 The recommendation also mentions the need of reducing the intake of ultraprocessed food, which is often found in the western and unhealthy patterns.

A diet rich in vitamins, minerals, fibers and antioxidants can stimulate the immune system and detoxification of enzymes, improve cholesterol synthesis, modulate hormone metabolism, and stimulate antioxidant defenses. 17 Besides, some studies link the intake of healthy food with healthier life habits, such as regular exercise, which can result in weight adequacy. Thus, promoting a healthy lifestyle during prenatal consultations is an excellent strategy for adequate weight gain during pregnancy. 21

The study conducted by Wei et al., 5 found out that the richer in fruits pattern was positively correlated to GWG. However, observing other components of this pattern, it was also possible to find a high presence of Cantonese dessert (sugar, rice flour, honey, whole milk). The presence of this type of high caloric intake in the pattern could explain the positive correlation to GWG, in compliance with the other presented results.

Unhealthy dietary patterns consist mainly of sweets, refined cereal, fast foods, salty snacks, red meat, fries, sugar-sweetened beverages, and alcoholic drinks. 20 23 24 The intake of unhealthy dietary patterns throughout pregnancy might be associated with excessive GWG due to its unbalanced offer of energy, and macro and micronutrients, thus contributing to undesired outcomes such as inadequate fetal growth, excessive fat accumulation, and metabolic complications. 17

Although some studies have shown a relationship between dietary patterns and GWG, and food consumption is one of the main factors causing inadequate or excessive GWG, other aspects of it must be taken into consideration. Pregestational BMI, and genetic and environmental factors (involving for instance the offer of, access to, and availability of food, and the context capable of promoting and impairing physical activities), as well as regular exercise by women, can also influence the herein addressed process. Thus, a dietary pattern alone may not be able to make a pregnant women develop inadequate or excessive GWG, a fact that could explain studies that did not find associations or that had their associations weakened by the adjusted models.

In addition to the data found in relation to GWG, dietary patterns during pregnancy also seem to influence weight gain in the years following the baby delivery. A cohort study performed in Norway found out that the adherence to the New Nordic Diet resulted in lower postgestational BMI and lower weight gain in the following 8 years after child delivery when compared with women who had low adherence to this diet. 27 The New Nordic Diet consists in a dietary pattern similar to that of traditional, healthy, and *prudent* patterns (fruits, roots, cabbage, potatoes, oat porridge, whole grains, wild fish, game meat, berries, milk and water). 28 The study concluded that adherence to the Norwegian eating guidelines, or adherence to Nordic diet guidelines recommended to pregnant women, are associated with lower postpartum weight retention.

It is known that dietary patterns can change based on the country or on the assessed population; however, other studies have also associated unhealthy, *western* , and sugar/fat-rich patterns with negative outcomes throughout pregnancy, whereas healthy or *traditional* patterns presented the best health outcomes. Kibret et al. 9 performed a review and meta-analysis and found that dietary patterns based on high fruit intake are associated with reduced chances to reach adverse results throughout pregnancy.

Other studies highlighted the relationship between gestational dietary patterns and pregnancy outcomes besides GWG, such as fertility, gestational diabetes mellitus, fetal growth, depression symptoms and preterm birth. 25 29 30 Such findings point out the magnitude of unhealthy dietary pattern influence on mother/child health outcomes. Shin et al. 19 assessed data from the National Research in Health and Nutrition and found a connection between high intake of refined grains, fat, addition sugar, and low intake of fruits and vegetables during pregnancy and greater chances to develop gestational diabetes mellitus.

The limitations of this systemic review must be recognized. The number of studies selected for this review was not large (only eleven studies fell within its scope) and the design of primary studies. In addition, the selected articles did not contain the necessary subsidies for the preparation of a meta-analysis. The time frame used for the study can also be considered a limitation, not using the Embase database, since more studies could be performed before that and on other platforms. However, this study also has several strengths. It is recognized that observational studies can be greatly influenced by confounding factors, such as lifestyle and sociodemographic variables, which can vary among different cultures and countries. This information was taken into account to minimize the confusion bias.

## Conclusion

Gestational dietary patterns a posteriori-derived that present ultraprocessed components rich in fat and sugars seem to be associated with excessive GWG, while healthy and traditional dietary patterns have been associated with better maternal and child health conditions, such as adequate GWG, term birth, and babies with adequate birth weight. However, the scarcity of studies on this topic points out the need for further investigation about the subject. Findings in the present review reinforce the importance of providing nutritional assistance to pregnant women during gestation and highlight the role played by public health policies focused on food and nutrition to encourage adhesion to healthier dietary patterns, which contributes to better maternal and newborn health outcomes.
